# Effects of infant feeding type on auditory event-related potentials at 24 months of age

**DOI:** 10.1038/s41390-024-03641-2

**Published:** 2024-12-05

**Authors:** Cecilia Algarín, Teresa Murguia-Peniche, Sussanne Reyes, Steven S. Wu, Jennifer L. Wampler, Patricio Peirano

**Affiliations:** 1 Sleep and Functional Neurobiology Laboratory, Institute of Nutrition and Food Technology (INTA), University of Chile, Santiago, Chile; 2 Medical Sciences, Reckitt | Mead Johnson Nutrition Institute, Evansville, IN USA; 3 School of Medicine, Indiana University, Indianapolis, IN USA

## Abstract

**Background:**

Added bovine milk fat globule membrane (bMFGM) or bMFGM components in infant formulas may favor language development essential for cognitive maturation in early life. In this study, the influence of infant feeding type on language skills acquisition was investigated.

**Methods:**

Auditory event-related potentials (ERPs) were performed at ~4–6 (baseline) and 24 months of age in infants receiving: human milk, as a reference group (HM, *n* = 42) or randomized to standard infant formula (SF, *n* = 53) or similar formula with added bMFGM (EF, *n* = 48) through 12 months of age. Auditive stimuli included three syllables (two native, one nonnative) with frequent or infrequent repetition. The main outcome was P1 wave amplitude and latency.

**Results:**

At baseline no significant differences were detected in P1 amplitude and latency by feeding or feeding×stimuli. At 24 months, P1 amplitude significantly differed by feeding type (*P* = 0.02; EF lowest). P1 latency for feeding×stimuli significantly differed for infrequent native (*P* = 0.01; longer for SF vs EF, *P* = 0.007).

**Conclusion:**

Electrophysiological changes in ERPs at 24 months of age demonstrated differences by infant feeding type and suggested beneficial effects of formula with added bMFGM on connectivity involved in language perception development.

**Impact:**

- First study to evaluate the nutritive effects of bovine milk fat globule membrane (bMFGM) on auditory event-related potentials (ERPs).
- First demonstration of differential language development associated with infant feeding type and bMFGM using an age-appropriate, high-sensitivity, electrophysiological method.
- Electrophysiological changes detected in ERPs at 24 months of age suggested an effect of added bMFGM in infant formula (compared to standard formula) which promotes faster neuronal transmission.

## Introduction

The first 2 years of life are crucial for brain development and acquisition of cognitive and socio-emotional functions, including language development. Infants extract the structure of language over time using a complex system of statistical learning.^1,2^ When syllables occur in a certain order, infants older than 6 months of age are able to detect the subsequent syllable and create clusters by means of statistical patterns.^3^ Auditory evoked response has been used to characterize brain processes related to language progression. Brain structures produce small voltages in response to a stimulus that can be measured in real time^4^ as event related potentials (ERP). Because responses do not require active attention, ERP acquisition is well-suited for infant and early childhood studies. Typical ERP testing during infancy is performed with repetitive presentation of an auditory stimulus at high probability to produce a habituated response that will contrast with stimuli presented at lower probability and lesser frequency.^5^ Resulting ERP waves are recorded in precise time windows.^6^ The two most important waves described in infancy are the P1 wave at ~200 ms and the negative N2 wave between 200 and 400 ms. The P1 wave reflects attentional processing of a stimulus by specific areas of the cerebral cortex and is more consistent in the first year,^7,8^ thus, the present study focused on the identification and changes of the P1. Origin and interpretation of P1 has been widely studied; current consensus is that P1 primarily presents in infancy and represents an early perceptual encoding of language learning, in addition to more complex functions such as categorization, lexical and attention process.^9,10^

Early nutrition also impacts brain structure and function. The milk fat globule membrane (MFGM) is a complex phospholipid trilayer that surrounds fat droplets secreted into all mammalian milk, including both human milk and bovine milk.^11^ Links between dietary consumption of MFGM components, including gangliosides (involved in neurogenesis), sphingomyelin (role in myelination), and phospholipids (component of neuronal membrane), and central nervous system myelination have been reported in preclinical rat^12^ and piglet models.^13^ For example, genes related to hippocampus development were upregulated and T-maze performance improved in newborn rats receiving dietary bovine (bMFGM).^14^ In infants, increased volume and rate of brain myelination in the first six months of age has been associated with receiving infant formula enriched with bMFGM components, including sphingomyelin.^15,16^ Dietary bMFGM has also been associated with beneficial effects on behavior^17^ and cognitive function.^18–21^ In a previous randomized controlled trial infants receiving formula with added bMFGM and bovine lactoferrin through 12 months of age had an accelerated neurodevelopmental profile at 12 months and improved language subcategories at 18 months of age^20^ as well as higher IQ in a follow-up study at 5.5 years of age.^21^ Infant formulas with added bMFGM have been clinically demonstrated to support infant growth and tolerance.^19,20,22–26^

In the current study, auditory ERPs at 24 months of age were evaluated as indirect indicators of myelination and circuit formation necessary for language abilities development in participants who were randomized to receive infant formula with or without added bMFGM through 12 months of age or received human milk as part of the reference group enrolled over the same study period.^26,27^

## Material and methods

### Sample and methods

The prospective, randomized, double-blind, controlled Chilean infant Nutrition Trial (ChiNuT) (ClinicalTrials.gov: https://clinicaltrials.gov/NCT02626143) was conducted between 2016 and 2018 in Santiago, Chile to evaluate growth through 24 months of age as the primary objective.^26,27^ Growth, body composition,^26^ micronutrient, metabolic, and inflammatory biomarkers^28^ through 24 months of age were previously reported. All recruitment, inclusion/exclusion criteria, randomization, and study formula information has been described previously.^27^ Briefly, eligible infants were randomized to receive one of two routine cow’s milk-based study formulas: (1) a standard formula (SF) or (2) a similar investigational formula (EF) that had an added bMFGM ingredient (whey protein-lipid concentrate, 5 g/L; Lacprodan® MFGM-10, Arla Foods Ingredients P/S, Denmark) from approximately 120 days of age through 12 months of age. Both study formulas had a prebiotic blend of polydextrose (PDX) and galactooligosaccharides (GOS) (4 g/L) and each study formula had per 100 kcal: docosahexaenoic acid (DHA) at 17 mg, arachidonic acid (ARA) at 25 mg, protein at 1.9 g, and iron at 1.2 mg.^27^ Infants exclusively receiving mother’s own milk were registered and assigned to a human milk reference group (HM). Participants from all study feeding groups were invited to participate in the cognitive assessments at the Sleep and Neurofunctional laboratory (INTA) until the pre-defined subgroup of approximately 150 participants was reached. Initial auditory ERPs were collected at baseline (as soon as possible following study entry) and at 24 months of age as a key neurodevelopmental outcome.

### Stimuli

All stimuli were presented using an oddball paradigm with EPrime® software (Psychology Software Tools, Inc.) amplified to a calibrated sound pressure level (SPL) of 60 dB. Sounds were in free-field to participants via left and right speakers attached to opposite walls of a sound-attenuated and electrically shielded room. The stimuli were consonant–vowel (CV) syllables which accomplished two functions. First was habituation, reached when a stimulus is presented with great frequency. For this study, the “frequent stimulus” was a CV syllable “ta”. Two stimuli presented less frequently, each termed “infrequent stimulus”, were the CV syllables “da” and “sha”. Second, the grade of familiarization with the habitual language was evaluated. Thus, two native language sounds “ta” and “da” were evaluated vs a non-native sound “sha”. In summary, the stimuli were: the frequent native (FN) stimulus (“ta” phonetically relevant in Spanish) and two deviants: an infrequent native (IN; CV syllable “da”) and an infrequent non-native (INN; “sha”), as has been previously described.^29^ A total of 1000 stimuli were presented (80% FN, 10% IN, and 10% INN). The stimulus onset-to-onset interval was 930 ms.

### ERP acquisition procedure

Each participant was seated on a parent’s laps in a comfortable chair that was positioned with its center equidistant from the face of the two speakers. Study site personnel engaged the participant’s attention with a puppet show or other toys. Alternatively, silent cartoons were played on tablet close to the participant to engage attention and minimize movement. ERPs were recorded from 128 scalp sites using a recording system with a geodesic sensor net (Electric Geodesic, Inc., Eugene, Oregon). The vertex was used as the on-line reference electrode. The signal was sampled at 1000 Hz and bandpass filtered at 0.1 to 100 Hz. After recording, stimulus triggers were exported using NetStation 5.4 software and analyzed using signal processing software (BESA Research 6.1 BESA® GmbH, Germany). Signals were re-referenced off-line to an average (whole head) reference and bandpass filtered (0.1 to 10 Hz). The continuous EEG was segmented into epochs according to the stimulus type (FN, IN, INN), with the segment length being the same as each onset-to-onset interval. A 50-ms pre-stimulus segment was included for baseline correction. Noisy segments of data with excessive electromyographic signal (EMG) were rejected by visual inspection, and noisy channels were identified and rejected using a 2.5% probability threshold and visual inspection. A channel rejection threshold was set at <20% and rejected channels were interpolated using a spherical method. In addition, the inactive eye channel electrodes were rejected, resulting in a data matrix of 124 channels for each subject. For ERP averaging, continuous data was filtered with a 1 to 15 Hz Butterworth band pass and epoch of −1500 to 1500 ms around stimulus presentation (i.e., “time 0”). An artifact rejection criterion of ±500 µV was used to reject noisy epochs and the maximum percent rejected limit was set at <30%.

### ERP data processing

Electrodes and time windows of interest were chosen as described previously.^30^ P1 waves were identified visually in a cluster of six electrodes in the medial-central region and the surrounding left and right areas. The left electrode cluster included: Fcz, Left FCT C3, CP1 and Cz, and the right cluster included: Fcz, CP2, C4, Right FCT and Cz. Within each cluster, the most positive peak occurring between 50 and 200 ms post-stimulus onset was identified as P1. Mean amplitudes were computed as the average amplitude within each condition (FN, IN, INN). Peak latency was also obtained for each condition and component. The averaged data from these six electrodes was used for data analysis.

### Data analysis

Demographic variables were analyzed by study feeding group. Variables determined as significantly different by group were included as covariates in each corresponding analysis of P1 waves (at baseline, at 24 months of age, or in the longitudinal baseline vs 24 months dataset). Repeated measures of multivariate analysis of variance (ANOVA) were performed to examine the main effects and study feeding group interaction (HM, SF, and EF) for P1 waves (mean ± SE) by study timepoint (baseline and 24 months of age). The within-participant factor was the three different stimuli (FN, IN and INN) and left and right hemispheres (LH, RH) and timepoint in longitudinal analysis. Post–hoc paired *t*-tests were conducted using Bonferroni correction for multiple comparisons. During ERP collection and data analysis, researchers continued to be blinded to study allocation. The pre-established analytical model included evaluation of three study feeding groups in which the independent variable was the infant feeding type (i.e., study feeding group): SF and EF and HM. The dependent variables were the latency and amplitude responses to the auditory stimuli: FN, IN and INN. Responses were analyzed by hemisphere (RH vs LH). Following the main analysis, analysis was also performed using a post-hoc statistical model that included only study formula groups as independent variables to better understand differences in ERP data. Statistical analyses were conducted with SPSS software version 19.0 (SPSS Inc., Chicago, IL). All significance tests were two-tailed; a *p* < 0.05 was considered statistically significant.

## Results

### Study population

**Fig. 1:**
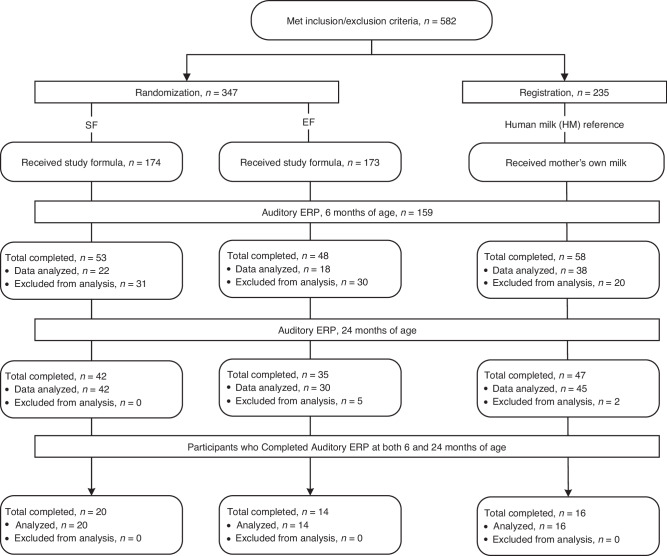
Study allocation. Study allocation. Study feeding group alllocation and study participant flow through 24 months of age.

Of 582 infants who met inclusion/exclusion criteria and were assigned to study feeding as previously described,^26^ parents of 159 participants agreed to participate in ERP acquisition (Fig. 1). Data from 60 participants fully met criteria to be included in statistical analysis at baseline; socio-demographic characteristics are described in Table 1. With the exception of a significant difference in age at study enrollment, no other group differences at baseline were detected. Data was excluded from analysis for the following reasons: did not show up (*n* = 1, 0.6%), outliers (amplitude or latency was greater than 2 SD; *n* = 12, 7.5%), did not meet requirements for analysis due to movement artifacts or fell asleep during more than 25% of the recording (*n* = 85, 53%). Demographics for participants who met (*n* = 60) or did not meet (*n* = 99) criteria for statistical analysis of ERP data (data not shown) were similar in composition.

**Table 1:** Social-demographic characteristics in participants with baseline ERP data (*n* = 60).

Characteristic	EF (*n* = 18)	SF (*n* = 22)	HM (*n* = 20)	*P*
Age. (days)	120 ± 27	123 ± 21	147 ± 18	0.001*
Total time formula received at baseline ERP (days)	89.67 ± 26.0	79.55 ± 28.7	n/a	0.25
Gestational age (weeks)	39.1 ± 1	38.9 ± 1.3	39.4 ± 1.1	0.39
Female, *n* (%)^a^	6 (33.3)	12 (54.5)	11 (55)	0.35
Weight at birth (g)	3507.2 ± 389.1	3310.8 ± 396.9	3392.7 ± 360.5	0.28
Length at birth (cm)	50.3 ± 1.8	49 ± 2	50.2 ± 2.3	0.08
Maternal age (years)	30.2 ± 6.2	28.2 ± 4.9	26.6 ± 5.2	0.12
Maternal BMI (kg/m^2^)	31.4 ± 5.1	30.6 ± 8.7	29.5 ± 5.8	0.66
Maternal education >12 years, *n* (%)^a^	7 (39%)	7 (32%)	11 (55%)	0.30
Family income ≥ US$8700 per year, *n* (%)^a^	9 (50%)	4 (18%)	5 (25%)	0.09

*n* = 60; Analysis of variance. mean ± SD.

*HM > SF (*P* = 0.002) and >EF (*P* = 0.001); age included as a covariant in the adjusted statistical analysis.

^a^Chi-square test or Fisher’s exact test.

Of the 159 participants who completed the baseline ERP acquisition, 129 (81.1.6%) returned for ERP acquisition at 24 months of age. Data from 122 participants fully met criteria to be included in statistical analysis and 7 were excluded (bad signal, *n* = 2; outlier responses, *n* = 5). Socio-demographic characteristics are described in Table 2. A higher proportion of participants met the criteria for analysis of data at 24 months in part because older participants appeared more collaborative and response to incentives increased with age, such as bubble play, and also by increased wake maintenance tone. Group sociodemographic characteristics were similar, with the exception of maternal education which was included as a covariable in the analysis.

**Table 2:** Socio-demographic characteristics in participants with ERP data at 24 months of age (*n* = 122).

Characteristic	EF (*n* = 35)	SF (*n* = 42)	HM (*n* = 45)	*P*
Age (days)	744 ± 24	744 ± 24	741 ± 30	0.85
Gestational age (weeks)	39.0 ± 1.0	38.8 ± 1.2	39.2 ± 1.2	0.41
Female, *n* (%)^a^	16 (45.7)	17 (40.5)	22 (48.9)	0.73
Weight at birth (g)	3489 ± 341.7	3481 ± 426.1	3383 ± 312.5	0.33
Length at birth (cm)	50.4 ± 1.6	50.2 ± 2.6	50.1 ± 2	0.74
Maternal age (years)	28.4 ± 5.8	28.1 ± 5.6	27.6 ± 5.8	0.80
Maternal BMI (kg/m^2^)	30.4 ± 6	30.1 ± 6.7	27.6 ± 4.7	0.06
Maternal education ≥12 years, *n* (%)^a,b^	28 (80%)	24 (57%)	18 (40%)	0.01
Family income ≥US$8700/year *n*, (%)^a^	15 (43%)	15 (36%)	24 (53%)	0.25

Analysis of variance, mean ± SD.

^a^Chi-square test.

^b^Included as a covariant in the adjusted statistical analysis.

### P1 amplitude (μV) and latency (ms) by feeding group, stimuli, and feeding group×stimuli at baseline and 24 months of age

#### Baseline

No significant differences in P1 wave amplitude or latency among study feeding groups were detected (Table 3). P1 amplitude (RH: 2.53 ± 0.14 vs LH: 2.69 ± 0.13; *P* = 0.053) and latency (RH: 189.56 ± 2.65 vs LH: 182.45 ± 2.67; *P* = 0.60) were similar between hemispheres. For responses to stimuli, P1 amplitude was significantly higher for IN (2.83 ± 0.15) vs FN (2.44 ± 0.14; *P* < 0.01) or vs INN (2.57 ± 0.14; *P* = 0.02). P1 wave latency was significantly longer for IN (192.7 ± 7.3) vs FN (180.4 ± 2.6; *P* < 0.01) or vs INN (184.8 ± 3.4; *P* = 0.02). Results could be interpreted as development of auditive discrimination capacity in this cohort of study participants, which is expected at this age in infants with typical cognitive development.^5^

**Table 3:** P1 wave amplitude and latency by feeding group and feeding group×stimuli at baseline.

P1	Parameter	EF (*n* = 18)	SF (*n* = 22)	HM (*n* = 20)	*P*, overall
Amplitude (μV)	Study Feeding	2.56 ± 0.25	2.55 ± 0.23	2.73 ± 0.25	0.87
	FN	2.44 ± 0.27	2.44 ± 0.24	2.43 ± 0.28	1.0
	IN	2.72 ± 0.28	2.74 ± 0.25	3.03 ± 0.28	0.81
	INN	2.51 ± 0.26	2.48 ± 0.24	2.71 ± 0.27	0.69
Latency (ms)	Study Feeding	186.6 ± 4.8	188.4 ± 4.31	183.07 ± 4.86	0.73
	FN	179.5 ± 5.0	181.8 ± 4.45	180.07 ± 5.02	0.90
	IN	185.8 ± 6.4	186.3 ± 5.75	182.5 ± 6.48	0.34
	INN	194.5 ± 5.1	197.1 ± 4.55	186.63 ± 5.13	0.91

Repeated measure ANOVA. Mean ± SE.

*FN* frequent native, *IN* infrequent native, *INN* infrequent non-native, *EF* Experimental formula, *SF* Standard formula, *HM* Human Milk.

#### 24 months of age

P1 amplitude was significantly different among study feeding groups (*P* = 0.02) (Table 4). For response to stimuli, P1 amplitude was significantly higher for IN (2.08 ± 0.07) vs INN (1.85 ± 0.06; *p* < 0.05) and vs FN (1.88 ± 0.06; *P* < 0.05). The interaction for feeding group×stimuli, demonstrated P1 amplitude for IN was significantly different (*P* = 0.02), driven by smaller amplitude in the EF.

**Table 4:** P1 wave amplitude and latency by feeding group and feeding group×stimuli at 24 months of age.

P1	Parameter	EF (*n* = 35)	SF (*n* = 42)	HM (*n* = 45)	*P*, overall
Amplitude (μV)	Study Feeding	1.76 ± 0.09	2.00 ± 0.08	2.08 ± 0.08	0.02^a^
	FN	1.67 ± 0.11	2.02 ± 0.10	1.97 ± 0.10	0.12
	IN	1.90 ± 0.13	2.03 ± 0.11	2.34 ± 0.11	0.02^b^
	INN	1.70 ± 0.11	1.92 ± 0.10	1.94 ± 0.10	0.15
Latency (ms)	Study Feeding	118.7 ± 1.5	121.3 ± 1.5	116.6 ± 1.7	0.10
	FN	115.1 ± 2.2	116.9 ± 2.0	114.7 ± 2.0	0.70
	IN	120.2 ± 1.7	127.3 ± 1.5	125.2 ± 1.5	0.01^c^
	INN	114.4 ± 2.1	119.7 ± 1.8	116.4 ± 1.9	0.16

Repeated measure ANOVA, Adjusted mean ± SE.

*FN* frequent native, *IN* infrequent native, *INN* infrequent non-native, *EF* Experimental formula, *SF* Standard formula, *HM* Human Milk.

^a^Pairwise comparison: EF < HM, *P* = 0.008.

^b^Pairwise comparison: EF < HM, *P* = 0.03.

^c^Pairwise comparison: EF < SF, *P* = 0.007.

For P1 latency, no significant differences among study feeding groups were detected. P1 latency was significantly longer for IN (124.22 ± 0.92) vs FN (115.69 ± 1.19; *P* < 0.01) and INN (116.62 ± 1.12; *P* < 0.01). For feeding group×stimuli, P1 latency for IN was also significantly different (*P* = 0.01) driven by shorter latency for the EF vs SF group (*P* = 0.007). No feeding group×stimuli differences in P1 amplitude or latency for FN or INN were detected. These results are consistent with lower perception of non-native language.^31^

### Change in P1 amplitude and latency, baseline vs 24 months of age

P1 wave amplitude and latency between baseline and 24 months of age was compared to explore maturation of brain related to perceived auditory stimuli. Participants who had ERP measures that met the criteria for analysis at both time points were included (*n* = 50); social demographic characteristics are described in Table 5.

**Table 5:** Socio-demographic characteristics in participants that had ERP data at both baseline and 24 months of age.

Characteristic	EF (*n* = 14)	SF (*n* = 20)	HM (*n* = 16)	*P*
Age first visit (days)	120 ± 27	123 ± 21	150 ± 18	0.001
Age second visit (days)	765 ± 18	765 ± 12	777 ± 24	0.214
Gestational age (weeks)	39.3 ± 0,9	38.9 ± 1.3	39.4 ± 1.2	0.389
Birth weight (g)	3547.4 ± 318.7	3291.9 ± 4123	3424.7 ± 330.3	0.137
Maternal age at birth	30.2 ± 6.0	28.4 ± 5.1	27.2 ± 5.6	0.331
Maternal BMI (kg/m^2^)	31.3 ± 5.5	30.0 ± 7.9	29.1 ± 5.3)	0.660
Female *n* (%)	9 (64.3)	8 (40.0)	7 (43.8)	0.403
Maternal education *n* (% < 12 years)^a^	8 (57.1)	14 (70.0)	8 (50.0)	0.445
Family income ≥ $8700/year *n* (%)^a,b^	7 (50%)	2 (10%)	4 (25%)	0.03

Analysis of variance, mean ± SD.

*HM* human milk, *SF* standard formula, *EF* experimental formula.

^a^Chi square or Fisher’s exact test.

^b^Included as a covariant in the adjusted statistical analysis.

**Fig. 2:**
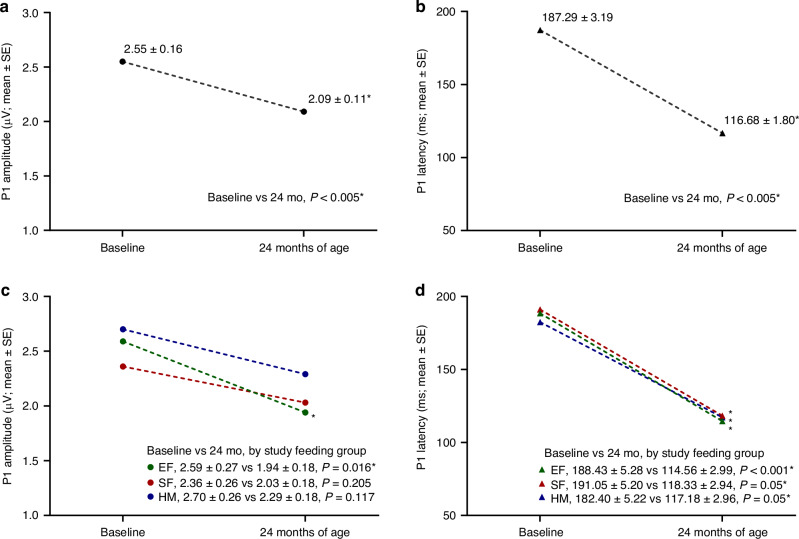
Developmental trajectory of P1 wave apmplitude and latency between baseline (~4 to 6 months of age) and 24 months of age. **a** P1 amplitude was significantly lower and (**b**) P1 latency was significantly shorter at 24 months of age vs baseline. **c** P1 amplitude among study feeding groups was significantly lower at 24 months of age vs Baseline for the EF group (*n* = 14); no significant age-related differences were detected for the SF (*n* = 20) or HM (*n* = 16) group. **d** P1 latency was significantly shorter at 24 months of age vs baseline for all study feeding groups.

Overall, P1 amplitude was significantly lower and P1 latency was significantly shorter at 24 months of age vs baseline as expected in children with typical brain development (Fig. 2a, b). By study feeding, P1 amplitude was significantly lower at 24 months of age vs baseline for the EF group; no significant age-related differences were detected for the SF or HM group (Fig. 2c). P1 latency was significantly shorter at 24 months of age vs baseline for all study feeding groups (Fig. 2d).

**Fig. 3:**
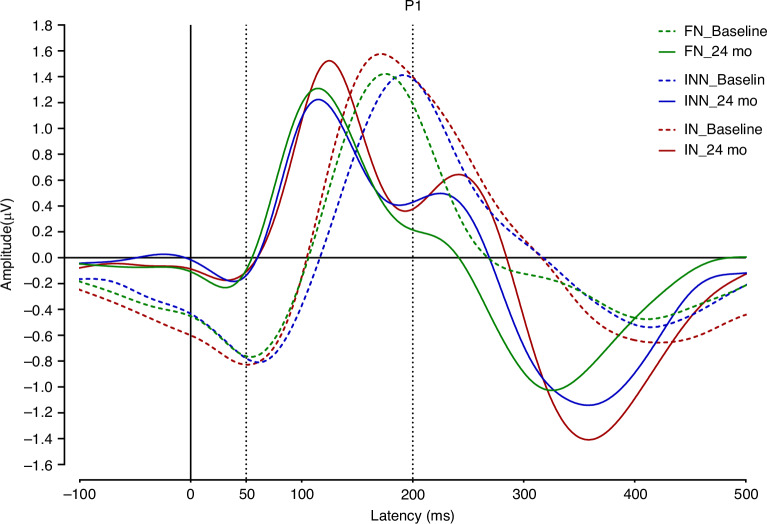
Age-related shifts in P1 amplitude and latency between baseline and 24 months of age. Representative waveforms by stimulus are shown for baseline (dotted lines) and 24 months of age (solid lines).

No significant differences were detected in the pattern of age-related changes in P1 amplitude and latency for FN, IN, or INN stimuli for baseline vs 24 months of age. To illustrate the age-related shifts in P1 amplitude and latency, representative waveforms by stimulus are shown for baseline (dotted lines) and 24 months of age (solid lines) (Fig. 3).

### Study formula groups-only at baseline and 24 months of age (post-hoc statistical model)

At baseline, P1 wave amplitude was similar between study formula groups (SF: 2.45 ± 0.26 vs EF: 2.44 ± 0.27; *P* = 0.98). For response to stimuli, a trend toward higher P1 amplitude for IN vs FN (2.63 ± 0.17 vs 2.30 ± 0.21; *P* < 0.06) was observed. P1 latency was also similar between study formula groups (SF: 187.35 ± 4.64 vs EF: 186.89 ± 4.77; *P* = 0.94).

At 24 months, P1 wave amplitude was similar between study formula groups (SF: 1.99 ± 0.07 vs EF: 1.79 ± 0.08; *P* = 0.08). For response to stimuli, P1 amplitude for IN vs INN was significantly higher (1.99 ± 0.08 vs 1.83 ± 0.07; *P* < 0.04). P1 wave latency was significantly shorter for the EF vs SF group (121.32 ± 1.47 vs 116.70 ± 1.61, *P* < 0.04).

## Discussion

In healthy term infants, we evaluated auditory ERPs at 24 months of age to characterize P1 wave amplitude and latency as an indirect measure of myelination and circuit formation necessary for language development. P1 wave amplitude in infants receiving bMFGM in infant formula compared to standard formula was smaller but did not reach significance (*P* = 0.052). For feeding×stimuli, P1 latency for IN was significantly shorter for infants receiving bMFGM in infant formula compared to the standard formula. These results in amplitude and latency may be translated as a faster perception to the auditory stimulus (example: native phoneme).

The initial assessment of ERPs at 4 to 6 months allowed us to establish the similar infant responses independent of the actual type of feeding. Cortical indices of sound localization mature in early infancy as early as two months of age^32^ and is evidence of the process development of cortical auditory perception and discrimination. In the current study, a consistent P1 wave in the frontocentral region was identified from ERPs initially acquired at baseline (around 4–6 months of age). Results of studies that have used a similar paradigm^33,34^ evidenced the habituation to the FN stimulus, which is expressed neurophysiologically as wave responses with lower amplitude and faster latency. Consistently, our results presented a lower FN response vs IN and INN stimuli response. In summary, baseline P1 wave analysis demonstrated the innate skill of infants to use auditory perception and the sensitivity to distinguish acoustic changes, which would express the ability to recognize familiar sounds and consequently, improve communication with the caregiver.^35,36^ As there were no differences detected by study feeding group, we may infer that participants presented similar neurophysiological responses at baseline.

In the current study, the amplitude for IN was larger compared to FN at 24 months of age, consistent with previous reports,^29,37^ suggesting participants had developed the ability to discriminate phonetically between the two native stimuli, which could represent an initial learning. P1 wave latency was significantly shorter for the FN stimulus suggesting the function of highly myelinated circuits is being activated by social interaction, which could represent one of the steps of language learning.^38^ Some other studies have reported no differences for P1 wave amplitude for FN vs IN at 24 months of age, possibly related to the use of other paradigms or other reasons yet-to-be identified.^34,38,39^ Whereas few studies have assessed how nutrition affects phonetic perception, variations in cognitive processes have been identified by diet. In the present study, at 24 months of age shorter latency for the IN stimuli in the MFGM group vs the standard formula group was demonstrated, suggesting better myelination in the former group. Other studies have compared different diet groups at different ages, for example, in a cohort of infants at 3–6 months of age, differences in language perception were related to feeding type (breastfeeding, cow’s milk-based formula, or soy-based formula).^40^ Shorter P1 latency and smaller P1 amplitude for deviant (IN) stimuli vs standard (FN) stimuli was detected for breastfed infants compared to infants receiving cow’s milk-based and soy-based formula^41^ which authors interpreted as indicators of more rapid encoding of acoustic information for breastfed infants versus other feeding groups.

Finally, to characterize age-related maturational changes and longitudinal changes in brain development associated with feeding type, the amplitude and latency of P1 wave responses were compared in participants who had ERP acquisition at both baseline and 24 months of age. As expected, participants at 24 months of age presented P1 wave characterized by lower amplitude and decreased latency.^42^

One of the challenges of designing infant formula is bringing the formulation closer to that of human milk, adding macronutrients in adequate proportions and micronutrients such as iron, zinc, calcium and essential fatty acids, to adequately support central nervous system development.^43^ This is particularly important to consider given the current study population of Chilean infants, where the breastfeeding rate in Chile is approximately 50% at 6 months of age.^44^ In addition, fatty acid composition human milk has variability according to the diet type of the country,^45^ the availability of food containing the specific nutrients, and the social and educational trends in which the mothers who are breastfeeding live.^46^

Strengths of this study include the large number of ERPs acquired and analyzed at 24 months of age and inclusion of a human milk group for comparison. Because no reference range for ERP outcomes is currently available, inclusion of a human milk group was important to study design and data analysis. However, only infants receiving formula could be randomized to the two study formula arms; the human milk group could not be randomized, limiting the ability to draw conclusions between the randomized groups and the human milk group as a reference. Consequently, post-hoc analysis was performed using a statistical model that included only the two study formula groups to focus on potential differences in ERP data between the randomized feeding groups. Consistent with the pre-specified main statistical model that included the human milk group, a similar pattern at baseline and 24 months of age was observed, which could be interpreted as faster auditive discrimination capacity at 24 months of age in participants who received infant formula with added bMFGM.

Additional limitations include the reduction of viable baseline data due to artifacts in ERPs acquired. Thus, the number of participants with data analyzed was smaller at baseline compared to 24 months. At 24 months several environment factors were included as covariables, but accounting for all factors is a well-known difficulty. Finally, an additional measured timepoint would provide more consistency in overall results, therefore follow up would be important to consolidate results.

## Conclusions

MFGM is a composite of many components that are involved in myelination, signaling and consequently in the circuitry formation including sphingomyelin, gangliosides, sialic acid, and cholesterol. From the original parent randomized controlled trial, we reported bovine MFGM in infant formula supported typical growth, body composition,^26^ and micronutrient, metabolic, and inflammatory biomarker status^28^ through 24 months of age. Additionally, in a separate study, infants receiving formula enriched with bovine MFGM and lactoferrin (compared to a standard formula with none added) had significantly higher cognitive scores at 12 months^19^ and significantly higher IQ scores at 5.5 years of age,^21^ supporting the potential beneficial effect of adding bovine MFGM to infant formula. Here we report at 24 months of age participants who received added bovine MFGM in formula presented a P1 wave response with shorter latency, compared to those receiving standard formula. These results could be interpreted as a higher grade of myelination and improved brain region connectivity, that in turn promoted greater efficiency to perceive a specific and well-characterized stimulus.

Because the impact of each environmental factor on infant nutrition cannot be accurately measured, a conservative approach to interpretation was taken. However, understanding and reporting on the functionality and health-based outcomes of adding bovine MFGM to infant formula is important as a significant proportion of infants in Chile and worldwide may receive partial or exclusive infant formula feeding when mother’s own milk is unavailable or breastfeeding is not chosen.

## Supplementary information

CONSORT-2010-Checklist-Algarin 2024 Pediatric Research submission

## Data Availability

The authors and study sponsor encourage and support the responsible and ethical sharing of data from clinical trials. De-identified participant data from the final research dataset used in the published manuscript may only be shared under the terms of a Data Use Agreement. Requests may be directed to: Jennifer.Wampler@reckitt.com.
